# Prognostic value of hepatocyte growth factor for muscle-invasive bladder cancer

**DOI:** 10.1007/s00432-021-03887-x

**Published:** 2022-01-08

**Authors:** Satoshi Katayama, Victor M. Schuettfort, Benjamin Pradere, Keiichiro Mori, Hadi Mostafaei, Fahad Quhal, Reza Sari Motlagh, Ekaterina Laukhtina, Nico C. Grossmann, Abdulmajeed Aydh, Pawel Rajwa, Frederik König, Pierre I. Karakiewicz, Martin Haydter, Marco Moschini, Mohammad Abufaraj, Yair Lotan, Richard K. Lee, Quoc-Dien Trinh, Eva Compérat, Jeremy Teoh, Yasutomo Nasu, Shahrokh F. Shariat

**Affiliations:** 1 Department of Urology, Comprehensive Cancer Center, Vienna General Hospital, Medical University of Vienna, Währinger Gürtel 18-20, 1090 Vienna, Austria; 2 Department of Urology, Okayama University Graduate School of Medicine, Dentistry and Pharmaceutical Sciences, Okayama, Japan; 3 Department of Urology, University Medical Center Hamburg-Eppendorf, Hamburg, Germany; 4 Department of Urology, The Jikei University School of Medicine, Tokyo, Japan; 5 Research Center for Evidence Based Medicine, Tabriz University of Medical Sciences, Tabriz, Iran; 6 Department of Urology, King Fahad Specialist Hospital, Dammam, Saudi Arabia; 7 Men’s Health and Reproductive Health Research Center, Shahid Beheshti University of Medical Sciences, Tehran, Iran; 8 Institute for Urology and Reproductive Health, Sechenov University, Moscow, Russia; 9 Department of Urology, University Hospital Zurich, Zurich, Switzerland; 10 Department of Urology, King Faisal Medical City, Abha, Saudi Arabia; 11 Department of Urology, Medical University of Silesia, 41-800 Zabrze, Poland; 12 Cancer Prognostics and Health Outcomes Unit, University of Montreal Health Centre, Montreal, Canada; 13 Department of Urology, Landesklinikum Wiener Neustadt, Vienna, Austria; 14 Department of Urology and Division of Experimental Oncology, Urological Research Institute, Vita-Salute San Raffaele University, 20132 Milano, Italy; 15 Division of Urology, Department of Special Surgery, The University of Jordan, Amman, Jordan; 16 Department of Urology, University of Texas Southwestern, Dallas, TX USA; 17 Department of Urology, Weill Cornell Medical College, New York, NY USA; 18 Division of Urological Surgery, Brigham and Women’s Hospital, Harvard Medical School, Boston, USA; 19 Department of Pathology, Medical University of Vienna, Vienna, Austria; 20 Department of Surgery, S.H. Ho Urology Centre, The Chinese University of Hong Kong, Hong Kong, China; 21 Department of Urology, Second Faculty of Medicine, Charles University, Prague, Czech Republic; 22 Karl Landsteiner Institute of Urology and Andrology, Vienna, Austria

**Keywords:** HGF, MIBC, Survival, Biomarker, Non-organ confined, Preoperative

## Abstract

**Purpose:**

The HGF/MET pathway is involved in cell motility, angiogenesis, proliferation, and cancer invasion. We assessed the clinical utility of plasma HGF level as a prognostic biomarker in patients with MIBC.

**Methods:**

We retrospectively analyzed 565 patients with MIBC who underwent radical cystectomy. Logistic regression and Cox regression models were used, and predictive accuracies were estimated using the area under the curve and concordance index. To estimate the clinical utility of HGF, DCA and MCID were applied.

**Results:**

Plasma HGF level was significantly higher in patients with advanced pathologic stage and LN metastasis (*p* = 0.01 and *p* < 0.001, respectively). Higher HGF levels were associated with an increased risk of harboring LN metastasis and non-organ-confined disease (OR1.21, 95%CI 1.12–1.32, *p* < 0.001, and OR1.35, 95%CI 1.23–1.48, *p* < 0.001, respectively) on multivariable analyses; the addition of HGF improved the predictive accuracies of a standard preoperative model (+ 7%, *p* < 0.001 and + 8%, *p* < 0.001, respectively). According to the DCA and MCID, half of the patients had a net benefit by including HGF, but the absolute magnitude remained limited. In pre- and postoperative predictive models, a higher HGF level was significant prognosticator of worse RFS, OS, and CSS; in the preoperative model, the addition of HGF improved accuracies by 6% and 5% for RFS and CSS, respectively.

**Conclusion:**

Preoperative HGF identified MIBC patients who harbored features of clinically and biologically aggressive disease. Plasma HGF could serve, as part of a panel, as a biomarker to aid in preoperative treatment planning regarding intensity of treatment in patients with clinical MIBC.

**Supplementary Information:**

The online version contains supplementary material available at 10.1007/s00432-021-03887-x.

## Introduction

Neoadjuvant chemotherapy, whenever possible, followed by radical cystectomy (RC) and lymphadenectomy is the standard of care for muscle-invasive bladder cancer (BCa) (Witjes et al. 2021). Despite efforts to completely excise cancer cells, nearly half of muscle-invasive BCa patients treated with RC alone succumbed to their disease within 5 years; patients with locally advanced disease and/or lymph node (LN) metastasis do significantly worse (Shariat et al. 2010). Given BCa’s aggressive biology, multimodality treatment, including perioperative systemic therapy, should be considered, especially for patients with presumably non-organ-confined (NOC) disease as they are most likely to harbor micrometastases (Karakiewicz et al. 2006; Svatek et al. 2010). Although neoadjuvant chemotherapy (NAC) is a well-established strategy supported by randomized phase III trials (Grossman et al. 2003; Griffiths et al. 2011), reliable biomarker-based accurate preoperative or postoperative identification of patients harboring NOC BCa or those suffering from aggressive tumor growth is yet to be established (Xylinas et al. 2014; Shariat et al. 2007). Indeed, based on clinical stage, a significant proportion of muscle-invasive BCa are likely to be overtreated by NAC.

Hepatocyte growth factor (HGF), a stromal cell-derived cytokine, is a natural endogenous ligand for MET receptors (Moosavi et al. 2019). Activation of the HGF/MET signaling pathway driven by a ligand (HGF)-dependent or ligand-independent manner promotes cellular proliferation, angiogenesis, tumor aggressiveness, and invasion, thereby resulting in poor survival outcomes (Mukai et al. 2020). To date, several available HGF/MET biomarkers, including HGF, MET, and soluble MET (sMET), have been investigated for their diagnostic, predictive, and prognostic roles in various malignancies (Gupta et al. 2008; Vuong et al. 2018). Of these, circulating HGF has been shown to be associated with worse pathologic and survival outcomes by several studies, such as lung or colorectal cancer (Tsuji et al. 2017; Toiyama et al. 2009). In bladder cancer, however, few studies have investigated the impact of circulating HGF (Gohji et al. 2000; Wang et al. 2007), and due to limitations of their sample size and study design, the significance and clinical utility of blood levels of HGF for the prediction of worse pathologic or poor survival outcomes remains unclear. Therefore, the present study aimed to assess the prognostic ability and clinical utility of plasma levels HGF obtained preoperatively in muscle-invasive BCa patients treated with RC.

## Material and methods

### Patient selection

We conducted a retrospective multi-institutional study including patients who underwent RC and lymphadenectomy for clinically nonmetastatic BCa. The study was approved by each institutional review board. We identified 1036 patients treated with RC for BCa, of which 471 who were diagnosed preoperatively with non-muscle-invasive disease (≤ cT1) were excluded to minimize the risk of confounding factors. Thus, 565 patients were included in the final analyses. None of the patients received NAC or perioperative radiotherapy. The extent of lymphadenectomy and urinary diversion type were based on the surgeon’s discretion.

### Management and follow-up

Preoperative staging of patients was performed using computed tomography or magnetic resonance imaging. All surgeries were performed with curative intent. The stages and grades of all specimens were determined according to the 8th edition of the American Joint Committee on Cancer staging system and 1973 World Health Organization system, respectively. Lymphovascular invasion (LVI) was examined in all samples. Due to the retrospective nature of the study, there was no standardized follow-up schedule. Generally, follow-up visits were undertaken quarterly for two years, semiannually between the second and fifth year, and annually thereafter. Tumor recurrence was defined as local recurrence or distant metastasis on imaging. The cause of death was determined by the treating physician based on a chart review corroborated by the death certificate or death certificate alone.

### Hepatocyte growth factor

Plasma samples were collected after preoperative overnight fasting on the morning of the day of surgery. Blood was collected into a Vacutainer CPT (Becton Dickinson Vacutainer Systems) and centrifuged at room temperature for 20 min at 1500 × g. The top layer corresponding to the plasma was decanted using sterile transfer pipettes. The plasma was immediately frozen and stored at − 80 °C in polypropylene cryopreservation vials (Nalgene, Nalge Nunc). For quantitative measurements of HGF levels, we used a commercially available quantitative immunoassay (DHG00B, R&D Systems). Every sample was run in duplicate, and the mean was calculated for data analysis. Coefficient of variation between measurements was less than 10% in all cases.

### Statistical analyses

The median value of HGF was calculated as 512.3 pg/mL (interquartile range [IQR]: 364.6–674.6). Based on the median value, patients were divided into the low and high HGF groups. The differences between continuous and categorical variables in the group were evaluated using the Mann–Whitney *U* test and chi-squared test, respectively. NOC disease was defined as ≥ pT3 and/or N + disease. Logistic regression models were used to assess the associations between HGF and other established confounding factors for the prediction of LN metastasis, pT3/T4 disease, and NOC disease. The predictive accuracy of the models was calculated using the receiver operating characteristic curve-derived area under the curve (AUC). AUCs were compared between the models with or without HGF using DeLong’s test. Kaplan–Meier survival curves were used to analyze the association between HGF and recurrence-free survival (RFS), overall survival (OS), and cancer-specific survival (CSS). The log-rank test was used to determine statistical differences between the groups. Multivariable Cox regression models were used to assess the association between prognostic factors including HGF and survival outcomes including RFS, OS, and CSS. When logistic regression and Cox regression models were used, HGF was treated as a continuous variable. For multivariable regression analyses, the values of HGF were divided by 100 to make meaningful interpretations. The discriminative ability of the Cox regression models was evaluated using Harrell’s concordance index. Decision curve analysis (DCA) was applied to assess the net benefit of the models with added HGF within a clinically reasonable range of threshold probabilities. The magnitude value of HGF for clinical practice was estimated according to the minimal clinically important difference (MCID) as previously suggested (Hayes 2021). Statistical significance was set at *p* < 0.05. All tests were two-sided. Analyses were performed using R version 3.6.3 (R Foundation for Statistical Computing, Vienna, Austria) and Stata/MP 14.2 statistical software (Stata Corp., College Station TX, USA).

## Results

### Patient characteristics

The distribution between HGF and clinicopathologic characteristics is described in Table 1. The median age was 66 (IQR, 59–73) years. Positive soft tissue surgical margins were found in 51 (9%) patients, and adjuvant chemotherapy (AC) was administered to 117 (21%) patients. An elevated level of HGF was correlated with an increased probability of advanced pathologic tumor stage (*p* = 0.01), presence of LVI (*p* = 0.03), and LN metastasis (*p* < 0.001).

**Table 1:** Patient characteristics

	Total	Low HGF (< 512.3 pg/mL)	High HGF (> 512.3 pg/mL)	*P *value
	*N* = 565	*N* = 282	*N* = 283	
Age, median (IQR)	66 (59, 73)	66 (60, 72)	67 (59, 73)	0.3
Sex, no (%)				0.2
Male	441 (78)	227 (80)	214 (76)	
Female	124 (22)	55 (20)	69 (24)	
Blood transfusion, no (%)	146 (26)	79 (28)	67 (24)	0.2
Clinical grade, no (%)				
Grade 3	565 (100)	282 (100)	283 (100)	
Clinical stage, no (%)				0.5
cT2	498 (48)	244 (87)	254 (90)	
cT3	38 (6.7)	22 (7.8)	16 (5.7)	
cT4	29 (5.1)	16 (5.7)	13 (4.6)	
Pathologic grade, no (%)				0.13
Grade 1	43 (7.6)	26 (9.2)	17 (6.0)	
Grade 2	1 (0.2)	1 (0.4)	0 (0)	
Grade 3	521 (92)	255 (90)	266 (94)	
Pathologic stage, no (%)				**0.01**
pT0	43 (7.6)	26 (9.2)	17 (6)	
pTa	2 (0.4)	1 (0.4)	1 (0.4)	
pTis	40 (7.1)	27 (9.6)	13 (4.6)	
pT1	40 (7.1)	22 (7.8)	18 (6.4)	
pT2	152 (27)	85 (30)	67 (24)	
pT3	200 (35)	83 (29)	117 (41)	
pT4	88 (16)	38 (13)	50 (18)	
Positive STSM, no (%)	51 (9.0)	21 (7.4)	30 (11)	0.2
LVI, no(%)	217 (38)	96 (34)	121 (43)	**0.03**
Concomitant CIS, no (%)	319 (56)	160 (57)	159 (56)	0.9
Number of removed LN, median (IQR)	22 (13, 33)	22 (13, 33)	21.5 (12-34)	0.9
LN metastasis, no (%)	186 (33)	71 (25)	115 (41)	**<0.001**
Adjuvant chemotherapy, no (%)	117 (21)	49 (17)	68 (24)	0.05

Bold *P* values are considered statistically significant

*HGF* hepatocyte growth factor, *IQR* interquartile range, *STSM* soft tissue surgical margin, *LVI l*ymphovascular invasion,* CIS* carcinoma in situ, *LN* lymph node

### Association with pathologic outcomes

**Fig. 1:**
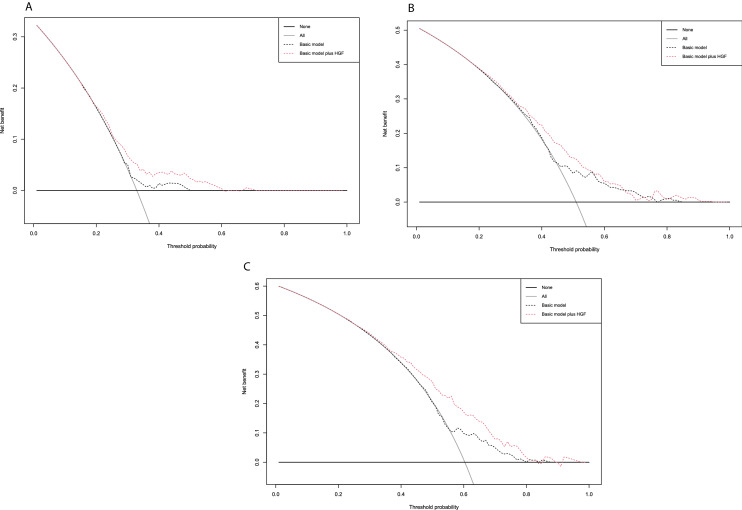
Decision curve analysis (DCA) for the net-benefit of hepatocyte growth factor (HGF) in the preoperative predictive model based on age, gender, clinical stage, and hepatocyte growth factor (HGF) for recurrence-free survival (RFS) (**A**), overall survival (OS) (**B**), and cancer-specific survival (CSS) (**C**) in muscle-invasive bladder cancer (MIBC) patients treated by radical cystectomy

**Fig. 2:**
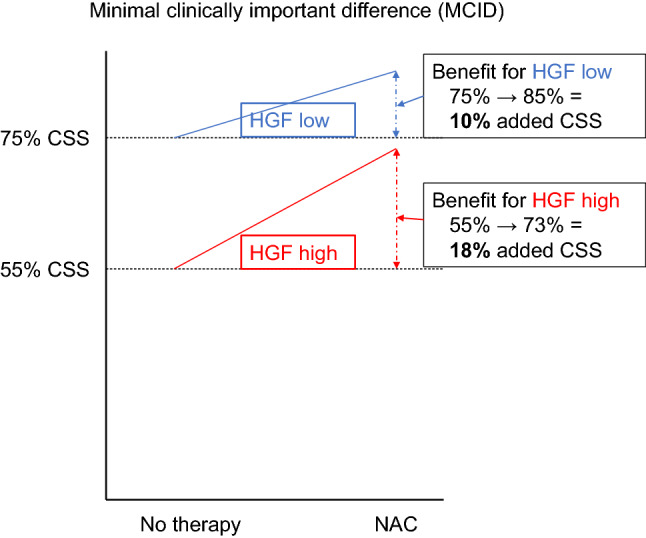
Minimal clinically important difference (MCID) for estimating the absolute magnitude of hepatocyte growth factor (HGF) when HGF is used to select patients who could benefit from neoadjuvant chemotherapy (NAC)

Logistic regression models for the prediction of worse pathologic outcomes are shown in Table 2. On multivariable logistic regression models that adjusted for the effects of established confounders, preoperative high HGF level was significantly associated with a higher risk of LN metastasis, pT3/T4 disease, and NOC disease (odds ratio [OR] 1.21, 95% confidence interval [CI] 1.12–1.32, *p* < 0.001; OR 1.23, 95% CI 1.13–1.34, *p* < 0.001; and OR 1.35, 95% CI 1.23–1.48, *p* < 0.001, respectively). Addition of HGF levels to the basic preoperative model, comprising age, sex, concomitant carcinoma in situ, and clinical stage, improved its discriminatory ability for the prediction of LN metastasis, pT3/T4 disease, and NOC disease by a statistically significant margin as measured by AUC values (+ 7%, + 6%, and + 8%, respectively). In DCA, given the clinically reasonable ranges of 30%-60%, addition of preoperative HGF level to the basic preoperative model allowed 53%, 47%, and 47% of patients with muscle-invasive BCa to improve by a net-benefit margin for the prediction of LN metastasis, pT3/T4 disease, and NOC disease, respectively (Fig. 1). Moreover, when modeled with NAC (applied HR of 1.66 (5)), 18% and 10% of additional patients with high and low HGF levels could benefit from an HGF measurement, respectively (Fig. 2), to ensure adequate therapeutic delivery. Subgroup analyses in cT2 patients revealed that preoperative HGF remained associated with a higher risk of LV metastasis, pT3/T4 disease, and NOC disease.

**Table 2:** Preoperative multivariable logistic regression models predicting lymph node involvement, pT3/T4 disease, and non-organ confined disease for patients with MIBC treated by RC

	Lymph node metastasis			pT3/T4 disease			Non-organ confined disease		
	Multivariable			Multivariable			Multivariable		
	OR	(95% CI)	*P *value	OR	(95% CI)	*P *value	OR	(95% CI)	*P *value
Age	1.00	0.98–1.01	0.6	1.03	1.01–1.05	**0.001**	1.03	1.01–1.04	**0.01**
Sex (ref. male)	1.49	0.97–2.27	0.07	1.15	0.76–1.76	0.5	1.26	0.81–1.97	0.3
Clinical stage (ref. cT2)									
cT3/cT4	1.44	0.83–2.46	0.2	3.56	2.00–6.63	**< 0.001**	3.33	1.79–6.58	**< 0.001**
HGF	1.21	1.12–1.32	**< 0.001**	1.23	1.13–1.34	**< 0.001**	1.35	1.23–1.48	**< 0.001**
AUC (95% CI)			**0.008**			**0.003**			**< 0.001**
Without HGF	55	50–60		61	57–66		62	57–67	
With HGF	62	57–67		67	63–72		70	66–75	

Bold *P* values are considered statistically significant

*MIBC *muscle-invasive bladder cancer*, RC *radical cystectomy,* HGF *hepatocyte growth factor, *CIS* carcinoma in situ, *AUC* area under the curve, *OR* odds ratio

### Association with survival outcomes

**Fig. 3:**
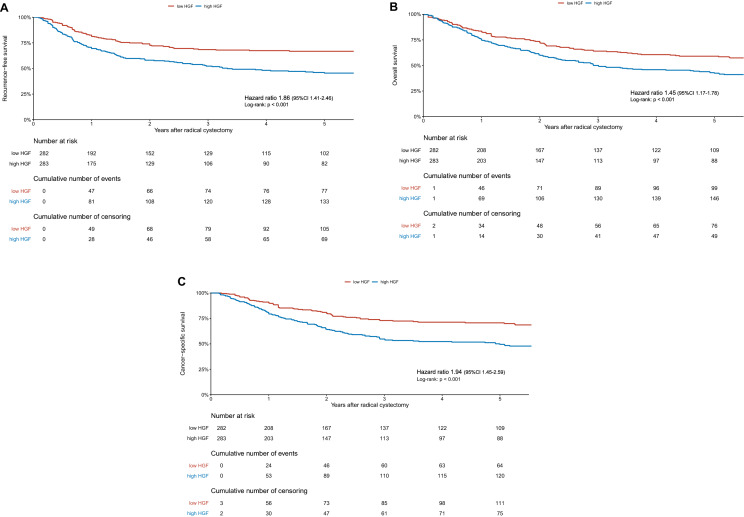
Kaplan–Meier analysis for recurrence-free survival (RFS) (**A**), overall survival (OS) (**B**), and cancer-specific survival (CSS) (**C**) in muscle-invasive bladder cancer (MIBC) patients treated by radical cystectomy according to the preoperative hepatocyte growth factor (HGF) level

During the median follow-up time of 28 (IQR, 10.5–105.5) months in patients alive, 216 (38%) patients experienced recurrence, 361 (64%) died from any causes, and 199 (35%) died from BCa. One-, two-, and five-year estimates for RFS, OS, and CSS rates were 76%, 66%, and 56%; 80%, 67%, and 51%; and 86%, 72%, and 60%, respectively. In Kaplan–Meier analyses, patients with preoperative levels of HGF above the median were at significantly higher risk of worse RFS, OS, and CSS compared to those with levels below the median (HR 1.86, 95% CI 1.41–2.46, *p* < 0.001; HR 1.45, 95% CI 1.17–1.78, *p* < 0.001; and HR 1.94, 95% CI 1.45–2.59, *p* < 0.001, respectively) (Fig. 3). On multivariable Cox regression analyses that adjusted for the effects of preoperative confounding factors, preoperative HGF remained associated with an increased probability of worse RFS, OS, and CSS (HR 1.16, 95% CI 1.10–1.23, *p* < 0.001; HR 1.09, 95% CI 1.04–1.14, *p* < 0.001; and HR 1.17, 95% CI 1.10–1.23, *p* < 0.001, respectively). Addition of HGF to a standard preoperative model improved the latter’s predictive accuracy by + 6% for RFS and by + 5% for CSS. On DCA, the addition of HGF slightly increased the net benefit for RFS (Supplementary Fig. 1). When adjusting for the effect of established postoperative variables in multivariable Cox regression analyses, HGF remained associated with an increased risk of RFS, OS, and CSS (HR 1.11, 95% CI 1.04–1.18, *p* < 0.001; HR 1.06, 95% CI 1.01–1.11, *p* < 0.001; and HR 1.11, 95% CI 1.04–1.18, *p* < 0.001, respectively). However, adding HGF to a standard postoperative model did not improve the latter’s predictive accuracy for any of survival endpoints (i.e., RFS, OS, and CSS) (Table 3). Subgroup analyses in cT2 patients revealed that preoperative HGF remained associated with an increased risk of RFS, OS, and CSS (Supplementary Tables 1 and 2).

**Table 3:** Preoperative and Postoperative Cox regression models predicting RFS, OS, and CSS for patients with MIBC treated by RC

	RFS					OS					CSS				
	Multivariable					Multivariable					Multivariable				
	HR	(95% CI)			*P *value	HR	(95% CI)			*P *value	HR	(95% CI)			*P *value
Preoperative model															
Age	1.01	1.00-1.03			0.13	1.04	1.03-1.06			**<0.001**	1.01	1.00-1.03			0.06
Sex (ref. male)	1.59	1.17-2.16			**0.003**	1.53	1.20-1.95			**<0.001**	1.74	1.27-2.37			**<0.001**
Clinical stage (ref. cT2)															
cT3/cT4	1.17	0.79-1.73			0.4	1.17	0.87-1.59			0.3	1.21	0.81-1.80			0.4
HGF	1.16	1.10-1.23			**<0.001**	1.09	1.04-1.14			**<0.001**	1.17	1.10-1.23			**<0.001**

C-index	Without HGF		With HGF			Without HGF			With HGF		Without HGF		With HGF		
	0.57		0.63			0.62			0.63		0.59		0.64		
Postoperative model															
Age	1.00	0.99-1.02			0.7	1.04	1.03-1.05			**<0.001**	1.01	0.99-1.02			0.3
Sex (ref. male)	1.56	1.14-2.13			**0.005**	1.51	1.18-1.93			**<0.001**	1.66	1.21-2.29			**0.002**
Pathologic stage (ref. pT0/pTa/pTis/pT1)															
pT2	1.72	0.86-3.42			0.12	1.30	0.89-1.89			0.2	1.69	0.82-3.49			0.2
pT3/T4	4.06	2.10-7.86			**<0.001**	2.25	1.55-3.28			**<0.001**	4.02	2.01-8.02			**<0.001**
Concomitant CIS	1.07	0.80-1.42			0.7	0.98	0.78-1.23			0.9	1.01	0.75-1.37			>0.9
Positive STSM	1.86	1.26-2.75			**0.002**	1.33	0.92-1.92			0.13	1.94	1.29-2.91			**0.001**
LVI	1.27	0.93-1.74			0.12	1.05	0.82-1.34			0.7	1.41	1.03-1.95			**0.034**
Pathologic grade (ref. Grade1/Grade2)															
Grade3	0.53	0.20-1.37			0.2	1.05	0.58-1.93			0.9	0.54	0.20-1.47			0.2
LN metastasis	2.32	1.66-3.23			**<0.001**	1.96	1.49-2.56			**<0.001**	2.17	1.54-3.06			**<0.001**
Adjuvant chemotherapy	0.85	0.61-1.19			0.3	0.86	0.65-1.14			0.3	0.88	0.63-1.25			0.5
HGF	1.10	1.03-1.16			**0.002**	1.05	1.00-1.10			**0.036**	1.10	1.04-1.17			**0.001**
C-index	without HGF			with HGF		without HGF		with HGF			without HGF			with HGF	
	0.75			0.75		0.72		0.72			0.76			0.77	

Bold *P* values are considered statistically significant

*RFS* recurrence-free survival, *OS* overall survival, *CSS* cancer-specific survival, *STSM* soft tissue surgical margin, *HGF* hepatocyte growth factor, *LVI* lymphovascular invasion, *LN* lymph node, *CIS* carcinoma in situ

## Discussion

In this study, we assessed the prognostic significance of preoperative HGF in a large multi-institutional cohort of patients with muscle-invasive BCa treated by RC. Higher preoperative HGF was an independent predictor of an increased risk of harboring more advanced pathologic stages, LN metastasis, and NOC disease. The increases in predictive accuracy compared to the standard preoperative model without HGF were 7% for LN metastasis (*p* < 0.001), 6% for pT3/T4 disease (*p* < 0.001), and 8% for NOC disease (*p* < 0.001). According to the DCA and MCID, approximately half of the patients had a benefit from HGF testing with moderate magnitude. In addition, an increased HGF level was a significant prognosticator of worse RFS, OS, and CSS in both preoperative and postoperative multivariable models that adjusted for confounders. These findings suggest that increased HGF level could preoperatively identify patients with features of aggressive BCa resulting in poor prognosis, leading to help refine clinical decision-making in terms of the adoption of perioperative systemic therapy and planning regarding the extent of surgery (e.g., lymphadenectomy template, nerve sparing etc.).

Despite a high level of evidence, only a third of patients who are candidates for NAC receive this strategy in the best of circumstances (McFerrin et al. 2020). Owing to the failure of accurate preoperative prediction based on useful pathologic information, several potential biomarkers or prediction models have been investigated to better identify preoperatively the patients with a high likelihood of advanced disease as they are the most likely to benefit from perioperative systemic therapy (NAC and/or AC) by a significant margins (Shariat et al. 2010, 2013; Moschini et al. 2017). As not all patients benefit from neoadjuvant systemic therapy, differentiating between those at low risk who could be treated with RC alone and those at high risk most likely to benefit from NAC (or other systemic therapy in the future) is of clinical necessity. The 5-year disease specific survival of low risk patients was greater than 80%, supporting the distinction of high risk and low risk muscle-invasive BCa. The presence of high risk features identifies patients with a poor prognosis who are most likely to benefit from NAC, while many of those with low risk disease can undergo surgery up front with good expectations and avoid chemotherapy associated toxicity (Moschini et al. 2017; Abufaraj et al. 2018). Subgroup analyses of cT2 patients found that preoperative HGF significantly improved the discriminative ability of worse pathologic and survival outcomes, implying that HGF value could help avoid unnecessary NAC. Moschini et al. previously confirmed that preoperative risk features can stratify patients with muscle-invasive BCa into differential risk groups regarding survival, suggesting that decision-making regarding neoadjuvant systemic therapy administration is likely to be improved by integrating clinical stage, LVI, variant histology, and hydronephrosis (Moschini et al. 2017). Abufaraj et al., however, demonstrated that detection of LVI is missed in a third of transurethral resection (TUR) specimens while variant histology (VH) seems more accurately identified, suggesting that LVI and VH on TUR specimen are important but not sufficient for risk stratification and decision-making. Therefore, there is a need for circulating cells that may reflect the micro metastasis status of the tumor at the moment.

Despite the availability of biomarker-based prediction tools, clinical decisions for muscle-invasive BCa treatment are still made without referring to any helpful prediction tools (Reardon et al. 2015; Grossman et al. 2019). There are several potential explanations for the low utilization of prediction tools in patient care. First, most prediction tools failed to be validated externally (Kluth et al. 2015). Additionally, many promising biomarkers or models have usually examined the presence of statistical significance and improved discrimination of the basic model, but could not have demonstrated its clinical utility worthy of changing the paradigm of standard of care. In the present study, we applied DCA and MCID to estimate the actual impact of HGF on our daily clinical practice. In the DCA, we found that approximately half of the patients were likely to receive an improvement in the predictive accuracy regarding the risk of worse pathologic outcomes, including LN metastasis or NOC BCa. In the MCID, we determined the magnitude of difference in the preoperative strategy by the use of plasma HGF as a biomarker. The added value of measuring HGF level was present limited; thus, in the context of clinical practice, despite the statistically significance, measuring only plasma HGF level may be insufficient to select or change the treatment strategies for MIBC patients treated with RC. HGF may be part of a comprehensive panel that reflects the biological and clinical behaviors of each tumor.

HGF, released by stromal cells, acts as a mediator of interactions between cancer cells and cancer-associated fibroblasts, which are fundamental to creating a tumor microenvironment that promotes tumor development and progression (Matsumoto et al. 1996; Spina et al. 2015). Binding of HGF to its receptor MET stimulates cancer cells through the phosphorylation of the HGF/MET signaling pathway, leading to downstream activation of multiple cytokines implicated in cellular motility, proliferation, angiogenesis, and invasive growth of cancer cells (Moosavi et al. 2019). Several diagnostic and prognostic biomarkers that represent aberrant activation of the HGF/MET pathway have been investigated. In bladder cancer, the major source of increased serum HGF levels is likely the tumor itself (Gohji et al. 2000); several studies have demonstrated the involvement of the HGF/MET pathway in the development of aggressive tumors. Urinary sMET was reported to be a useful diagnostic marker to distinguish patients with and without BCa. The values of tissue HGF and c-MET have also been shown to be associated with poor survival (Cheng et al. 2002; Miyata et al. 2009). Our findings, in accordance with previous studies, showed that increased HGF level was independently associated with an increased probability of worse RFS, OS, and CSS as well as that of worse pathologic outcomes. However, the performance of the postoperative prediction model for survival was weak, suggesting no benefit to HGF in the postoperative setting. This is likely because of the strong correlation between HGF and pathologic features.

Another potential use of HGF is the application of targeted therapeutic agents. The HGF/MET pathway has also been implicated in cellular survival and therapeutic resistance. Cabozantinib, a multikinase inhibitor of HGF/MET, VEGFR, and AXL, has been shown to prevent angiogenesis and invasive growth. With HGF/MET pathway being considered a key alternative after disease progress to therapies targeting the primary driver, the MET inhibitors have demonstrated promising results in combination with immune-checkpoint inhibitors for patients with advanced BCa (Apolo et al. 2020). Compared to MET amplification or mutations, the role of HGF in acquired resistance still remains to be determined (Guo et al. 2020). Better understanding of the HGF/MET axis in BCa might accelerate the establishment of HGF/MET inhibitors for BCa patients including the perioperative setting using a biomarker approach.

The present study has some limitations that are mainly inherent to retrospective data collection. The identification of actual impact of plasma HGF was hindered by the lack of standardized cut-off points. The lack of a standard template for lymphadenectomy and central pathologic reviews may have affected the evaluation of our intended outcomes, which may have limited our ability to predict them accurately. However, standard LN sampling may have limited the excision of LNs and detection of all positive LNs as much as possible. Administration of adjuvant chemotherapy and follow-up management were not standardized. Despite these limitations, we identified a prognostic value to preoperative HGF level in the preoperative setting. Further large controlled studies on HGF in BCa are warranted to explore its clinical utility as a prognostic biomarker and target for therapeutics to allow individualized treatment strategies of BCa.

## Conclusion

An increased level of HGF was associated with clinicopathologic features of aggressive disease, including LN metastasis or advanced pathologic stage. In the preoperative setting, the addition of HGF improved predictive accuracy for any NOC BCa by a statistically and prognostically significant margin and moderate magnitude; the same was true for prognostication of RFS and CSS in cT2-T4 patients treated with RC. This biomarker has potential to accurately identify patients who benefit from intensified management including NAC and/or extended lymphadenectomy in our clinical practice. Together with other biomarkers, it may allow for more precise care delivery for each muscle-invasive BCa patient.

## Supplementary Information

Below is the link to the electronic supplementary material.

Supplementary file1 (PDF 355 kb)

## Data Availability

Yes.
