# Supplementary material for: Prognostic value of hepatocyte growth factor for muscle-invasive bladder cancer
Source: J Cancer Res Clin Oncol. 2022 Jan 8;148(11):3091–102. doi: 10.1007/s00432-021-03887-x (PMC9508199; doi:10.1007/s00432-021-03887-x)

Supplementary Table1. Preoperative multivariable logistic regression models predicting lymph node involvement, pT3/T4 disease, and non-organ confined disease for cT2 patients with MIBC treated by RC

	**Lymph node metastasis**			**pT3/T4 disease**			**Non-organ confined disease**		
	Multivariable			Multivariable			Multivariable		
	OR	(95% CI)	*P* value	OR	(95% CI)	*P* value	OR	(95% CI)	*P* value
**Age**	1.00	0.98-1.02	>0.9	1.03	1.01-1.05	**0.004**	1.03	1.01-1.05	**0.01**
**Sex** (ref. male)	1.25	0.78-1.97	0.3	1.08	0.70-1.69	0.7	1.18	0.74-1.88	0.5
**HGF**	1.21	1.11-1.33	**<0.001**	1.22	1.12-1.33	**<0.001**	1.34	1.22-1.48	**<0.001**
**AUC** (95% CI)			**0.003**			**0.005**			**<0.001**
without HGF	51	45-56		57	52-62		57	52-62	
with HGF	62	56-67		64	59-69		68	64-73	

Bold P values are considered statistically significant.

MIBC muscle invasive bladder cancer, *RC* radical cystectomy, *HGF* hepatocyte growth factor, *CIS* carcinoma in situ, *AUC* area under the curve, *OR* odds ratio

Supplementary Table 2. Preoperative Cox regression models predicting RFS, OS, and CSS for cT2 patients with MIBC treated by RC

		**RFS**			**OS**			**CSS**		
		Multivariable			Multivariable			Multivariable		
		HR	(95% CI)	*P* value	HR	(95% CI)	*P* value	HR	(95% CI)	*P* value
**Preoperative model**	**Age**	1.01	1.00-1.03	0.2	1.04	1.03-1.06	**<0.001**	1.01	1.00-1.03	0.11
	**Sex** (ref. male)	1.53	1.09-2.14	**0.014**	1.44	1.11-1.88	**0.007**	1.67	1.18-2.35	**0.004**
	**HGF**	1.17	1.10-1.24	**<0.001**	1.09	1.04-1.14	**<0.001**	1.17	1.10-1.24	**<0.001**
	**C-index**	without HGF		with HGF	without HGF		with HGF	without HGF		with HGF
		0.56		0.62	0.61		0.63	0.58		0.63

Bold P values are considered statistically significant.

RFS recurrence-free survival, *OS* overall survival, *CSS* cancer-specific survival, *HGF* hepatocyte growth factor,

Supplementary Figure 1A

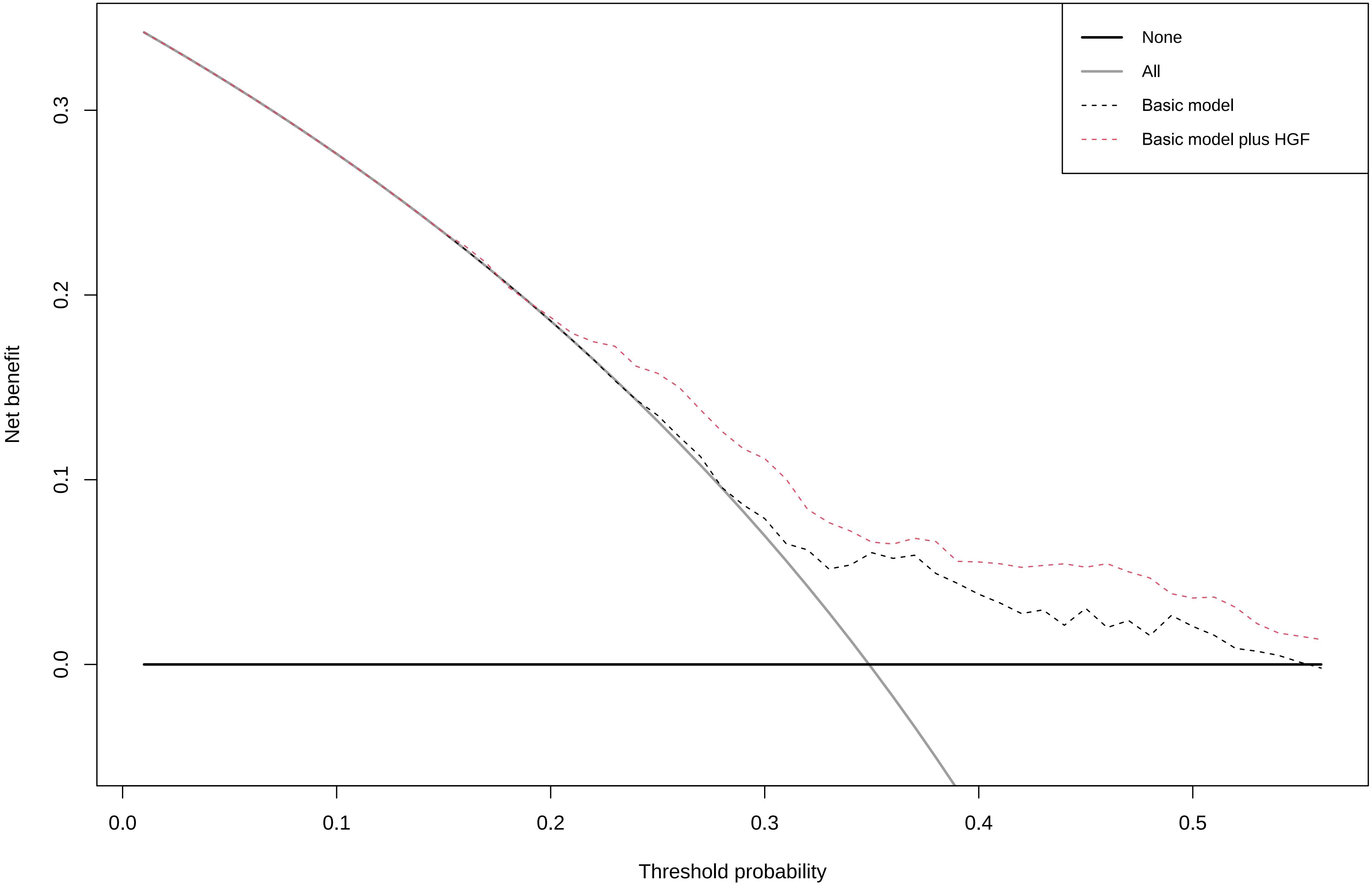

Supplementary Figure 1B

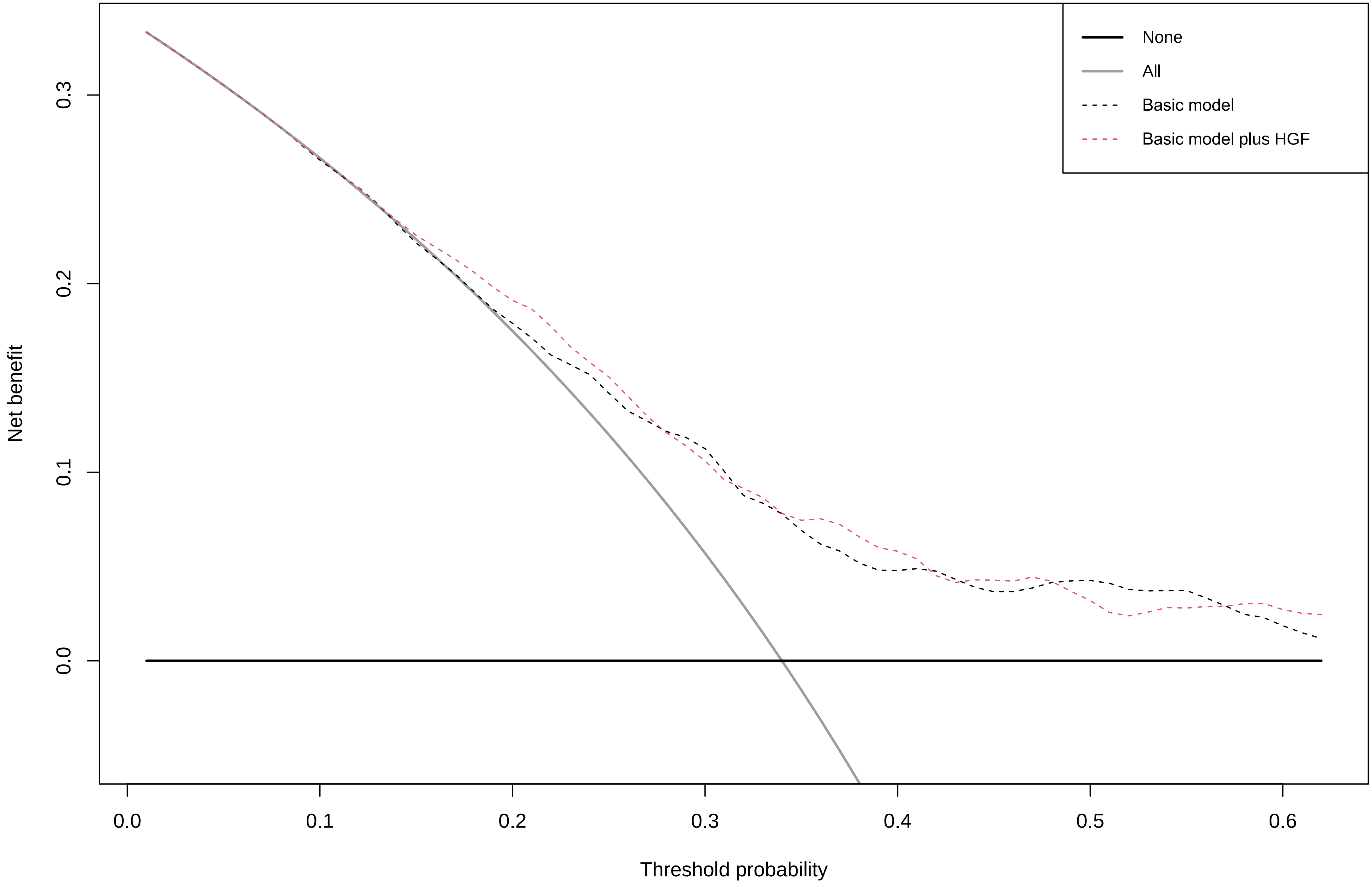

Supplementary Figure 1C

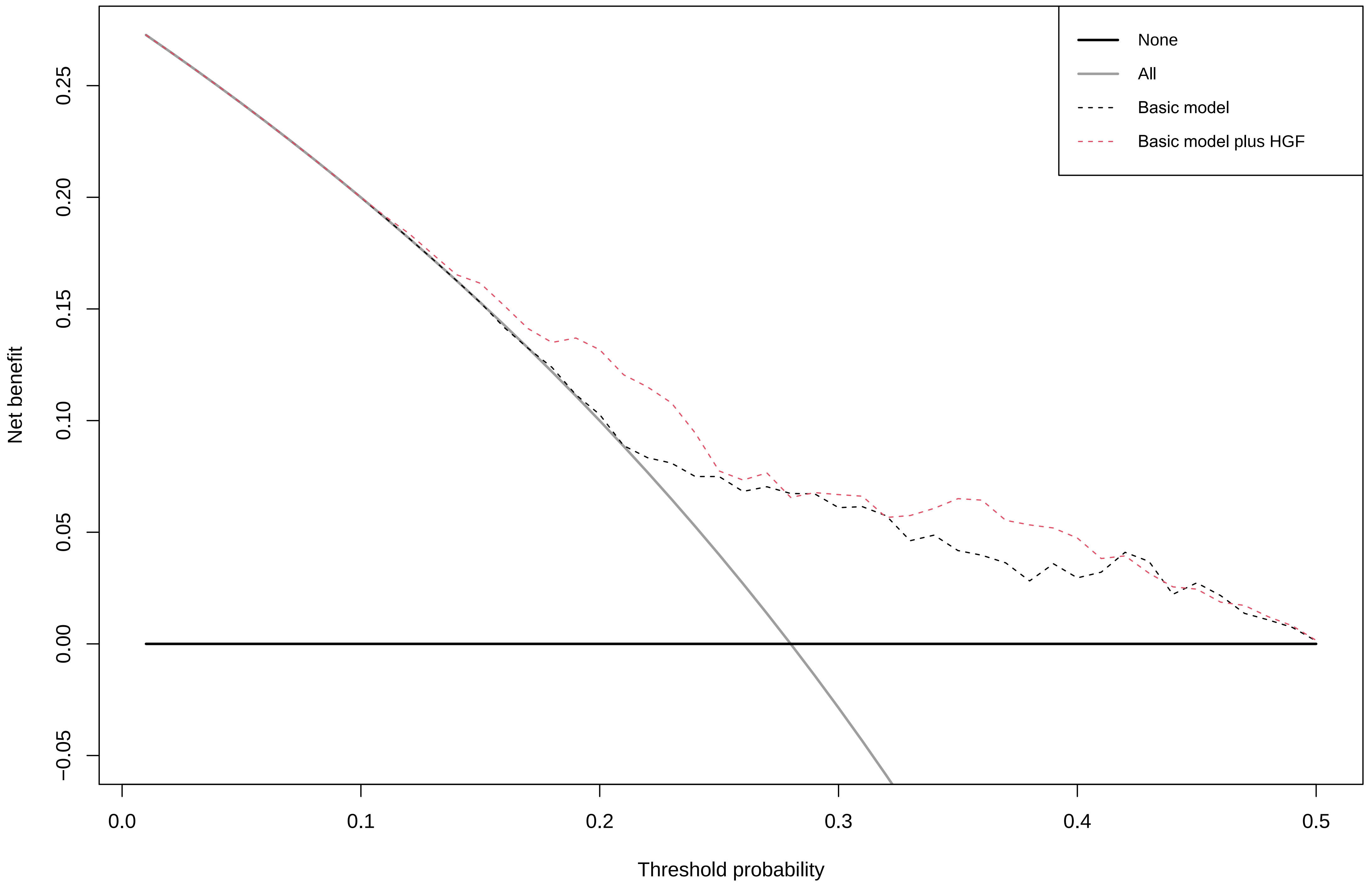

Supplement: Supplementary file 1 — Supplementary file1 (PDF 355 kb) [file 432_2021_3887_MOESM1_ESM.pdf]
